# Value of Literature Review to Inform Development and Use of Biologics in Juvenile Idiopathic Arthritis

**DOI:** 10.3389/fped.2022.909118

**Published:** 2022-06-21

**Authors:** Klervi Golhen, Carolyn Winskill, Cynthia Yeh, Nancy Zhang, Tatjana Welzel, Marc Pfister

**Affiliations:** ^1^Pediatric Pharmacology and Pharmacometrics, University Children’s Hospital Basel (UKBB), University of Basel, Basel, Switzerland; ^2^Integrated Drug Development, Certara LP, Princeton, NJ, United States; ^3^Pediatric Rheumatology, University Children’s Hospital Basel (UKBB), University of Basel, Basel, Switzerland

**Keywords:** randomized controlled trials, systematic review, efficacy, safety, juvenile idiopathic arthritis, bDMARDs, literature review, JAK inhibitors

## Abstract

**Background:**

Juvenile idiopathic arthritis (JIA) is one of the most common pediatric inflammatory rheumatic diseases (PiRDs). Uncontrolled disease activity is associated with decreased quality of life and chronic morbidity. Biologic disease-modifying antirheumatic drugs (bDMARDs) and Janus kinase inhibitors (JAKi) have considerably improved clinical outcomes. For optimized patient care, understanding the efficacy-safety profile of biologics in subgroups of JIA is crucial. This systematic review based on published randomized controlled trials (RCTs) aims to assess efficacy and safety data for bDMARDs and JAKi with various JIA subgroups after 3 months of treatment.

**Methods:**

Data for American College of Rheumatology (ACR) pediatric (Pedi) 30, 50, and/or 70 responses after 3 months of treatment were selected from RCTs investigating bDMARDs or JAKi in JIA according to predefined inclusion/exclusion criteria. Treatment and control arms were compared by calculating risk ratios (RRs) with 95% confidence intervals (CIs), and proportions of overall, serious adverse events (AEs) and infections were analyzed. Forest plots were generated to summarize efficacy and safety endpoints across studies, JIA subgroups, and type of biologics.

**Results:**

Twenty-eight out of 41 PiRD RCTs investigated bDMARD or JAKi treatments in JIA. 9 parallel RCTs reported ACR Pedi 30, 50, and/or 70 responses 3 months after treatment initiation. All treatment arms showed improved ACR Pedi responses over controls. RRs ranged from 1.05 to 3.73 in ACR Pedi 30, from 1.20 to 7.90 in ACR Pedi 50, and from 1.19 to 8.73 in ACR Pedi 70. An enhanced effect for ACR Pedi 70 was observed with infliximab combined with methotrexate in PJIA vs. methotrexate monotherapy. A slightly higher risk of gastrointestinal AEs and infections was observed with treatment arms compared to placebo or methotrexate monotherapy.

**Conclusion:**

Investigated bDMARDs and JAKi showed superior treatment responses compared to controls after 3 months of treatment, which were more pronounced in ACR Pedi 50 and 70 than in ACR Pedi 30. Higher susceptibility to infections associated with bDMARDs or JAKi vs. control arms must be weighed against efficacious treatment of the underlying disease and prevention of disease-related damage. Additional RCTs are warranted to further inform development and utilization of biologics in JIA.

## Introduction

“Pediatric inflammatory rheumatic diseases” (PiRDs) is an umbrella term for chronic inflammatory conditions affecting infants, children, and adolescents. Juvenile idiopathic arthritis (JIA) is one of the most common PiRDs and was originally defined as chronic arthritis with onset before the 16th birthday and persisting for at least 6 weeks after exclusion of other known conditions (1, 2). In the proposed updated classification criteria, JIA is defined as an inflammatory disease that begins before the 18th birthday and persists for at least 6 weeks after other conditions have been excluded (3). According to the International League of Associations for Rheumatology (ILAR), JIA encompasses seven subgroups classified as systemic JIA (sJIA), oligoarticular JIA (OJIA), rheumatoid factor-positive (RF +) or negative (RF–) polyarticular JIA (PJIA), enthesitis-related JIA (ERA), psoriatic arthritis, and undifferentiated arthritis (1). A vast majority of patients with JIA is affected by uveitis (JIA-uveitis). JIA-uveitis is a frequent and devastating extra-articular manifestation of JIA that commonly affects children aged 3–7 years (4). Both JIA and JIA-uveitis are associated with risks of chronic morbidity, loss of functionality, ocular sequelae, and vision loss, as well as decreased health-related quality of life in the case of uncontrolled disease activity over time (5–7). Thus, early efficacious and safe treatment is crucial.

In recent years, several cytokine-targeting/neutralizing treatments such as biologic disease-modifying antirheumatic drugs (bDMARDs) and Janus kinase (JAK) inhibitors have been developed. In addition, core set criteria have been developed in pediatric rheumatology to assess standardized disease activity and treatment responses. The assessment of changes in defined core set criteria over time enables clinicians to determine whether patients demonstrate significant clinical improvement or worsening in their disease, and guides clinicians in disease management. In JIA, the definition of improvement in response to a treatment can be assessed with the American College of Rheumatology (ACR) pediatric (Pedi) responses (8). These criteria are based on six core outcome variables for JIA: physician global assessment (PGA) of disease activity (measured on a 0–10 visual analog scale (VAS) with 0 = no activity and 10 = maximum activity); parent/patient assessment (PPGA) of overall wellbeing (10-cm VAS); functional ability; number of joints with active arthritis (defined as joint effusion or limitation of motion accompanied by heat, pain, or tenderness); number of joints with limited motion; and erythrocyte sedimentation rate (9). An ACR Pedi 30 response is defined as at least a 30% improvement from baseline in three out of six variables with no more than one remaining variable worsening by > 30% (9). The ACR Pedi criteria have been adapted for use in clinical trials in sJIA by adding the demonstration of the absence of spiking fever to the six core set variables (9). Given the increasing clinical demand of high response levels, ACR Pedi 50, 70, 90, and 100 levels of response expanded the disease activity assessment scale. Malattia et al. have shown that long-term ACR Pedi 90 responses are important to avoid articular damage (10). In addition, treat-to-target (T2T) approaches have become more and more important in rheumatic disease management (11). The T2T approach describes the principle of selecting treatment type and dose with adjustments made according to assessed disease activity, with the aim of achieving no disease activity/lowest possible disease activity (referred to as the defined target). Disease activity and treatment responses are assessed regularly (usually every 3–6 months) to adjust treatment type and dose, as uncontrolled disease activity may severely compromise the patients and their families with negative implications on physical and mental health.

Within the past few years, highly effective bDMARDs and JAK inhibitors have been developed and approved, allowing patients to reach desired targets such as clinical remission or lowest achievable disease activity. However, in JIA the number of approved bDMARDs and JAK inhibitors is still limited compared to rheumatoid arthritis (RA) in adult rheumatology (12). This may lead to increased unlicensed and off-label use in daily practice to treat pediatric patients with high disease activity and unresponsiveness to approved bDMARDs and JAK inhibitors. Although off-label use does not mean unawareness, it is often of great concern to the families and their affected children (13). Data indicates that unlicensed and off-label drug use in children may increase the risk of dosing errors and adverse events (14–16). Higher risk of infections associated with bDMARDs or JAK inhibitors, with greater risk in case of overdosing, should be weighed against effective treatment allowing disease activity control and prevention of disease-related damage. In pediatric trials, several dosing regimens are commonly selected based on existing data from adult (17). However, there are noticeable differences between children with JIA and adults with RA at various levels such as disease and disease course, risk of comorbidities, pharmacokinetics and pharmacodynamics, and attainment of drug-free remission. As such, the aims of this systematic review are to provide insights regarding (i) efficacy with bDMARDs and JAK inhibitors in JIA after 3 months of treatment by analyzing ACR Pedi 30, 50, and 70 responses, and (ii) safety of bDMARDs and JAK inhibitors based on previously published randomized controlled trials (RCTs), with the goal to (iii) facilitate risk-benefit assessment and optimization of pediatric drug development and clinical practice.

## Methods

This systematic review was conducted based on the Cochrane Handbook for Systematic Reviews of Interventions and reporting items in the PRISMA statement (18, 19). This systematic review focused on comparing risk ratios (RRs) of ACR Pedi 30, 50, and 70 (efficacy endpoints) and incidence of adverse events (AEs) of interest (safety endpoints) between RCTs in JIA treated with bDMARDs or JAK inhibitors.

### Literature Search and Selection of Trials

**FIGURE 1:**
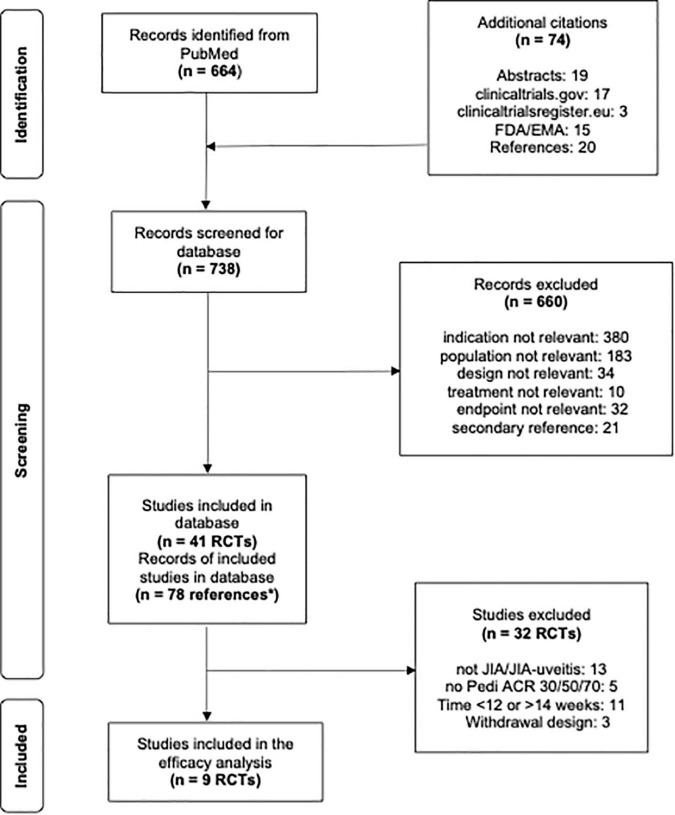
Flowsheet literature review and study selection for analysis. *n = 78 Correspond to multiple references to a single study, i.e., more than one journal article, clinicaltrials.gov and/or regulatory documents.

**FIGURE 2:**
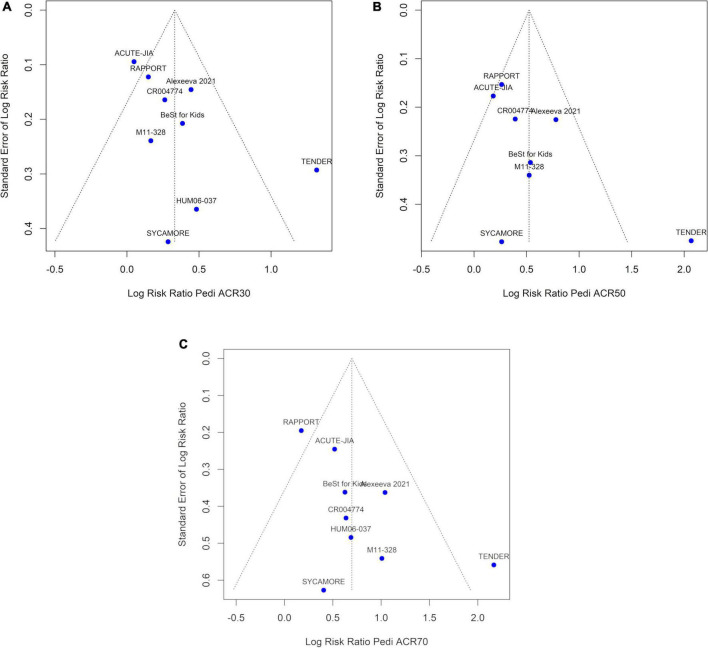
Funnel plots asymmetry tests, using data from **(A)** ACR Pedi 30 data **(B)** ACR Pedi 50 data **(C)** ACR Pedi 70 data, with log-risk ratios displayed on the horizontal axis.

This systematic review was performed in line with a previously conducted systematic literature search initially performed on July 26, 2020, in MEDLINE, ClinicalTrials.gov, and the EU Clinical Trials Register, with a sample size of ≥ 5 children with PiRD aged ≤ 20 years and treated with predefined bDMARDs and JAK inhibitors (12). On February 3, 2022, this literature search was updated in line with the review protocol (see Figure 1 and Supplementary Material) (12). When multiple references to a single study (i.e., more than one journal article, clinicaltrials.gov and/or regulatory documents) were available, data from one study was pulled from all available sources. Identified RCTs fulfilling the following inclusion criteria were included in the systematic review: (i) population: JIA or JIA-uveitis; (ii) treatment: abatacept, adalimumab, anakinra, baricitinib, belimumab, brodalumab, canakinumab, certolizumab, etanercept, golimumab, guselkumab, infliximab, ixekizumab, risankizumab, rilonacept, rituximab, sarilumab, secukinumab, tildrakizumab, tocilizumab, tofacitinib, upadacitinib, ustekinumab; (iii) outcomes: ACR Pedi 30, 50, or 70 responses; (iv) time points: 12–14 weeks (3 months) after treatment start. Studies that reported ACR Pedi responses before week 12 or after week 14 were excluded from analysis. Any non-bDMARD arms that were not designated as the control arm for a study (i.e., conventional DMARD arms) were also excluded from analysis. Withdrawal studies are enrichment designs, in which only responders to open-label treatment are randomized, therefore overestimating the treatment effect and affecting external validity of results. As such, withdrawal studies were excluded from efficacy analysis. Funnel plots of all included studies were performed to assess publication bias (Figure 2).

### Data Collection

Aggregate (summary)-level data was extracted for each included RCT. Study design (i.e., parallel or withdrawal), baseline demographic and clinical characteristics such as location, patient population, sample size, age criteria, and treatment, as well as efficacy and safety data were captured.

### Efficacy Data

The ACR Pedi 30, 50, and 70 responses at week 12–14 (i.e., after 3 months of treatment) were used to describe efficacy. The ACR Pedi responses were screened in line with the six core set variables defined by Giannini et al. (8). For sJIA, in line with previous clinical trials, the adapted ACR Pedi responses including absence of spiking fever were used. If multiple statistical analyses were reported (model-estimated proportions in addition to raw data), the raw data was selected.

### Safety Data

Each RCT included in the systematic review, including withdrawal designs (12 RCTs), was screened for safety data. A majority of studies reported safety data at the end of each study (e.g., after 3 or 6 months of treatment). Safety data of interest included (i) overall AEs, (ii) serious AEs (SAEs), (iii) overall infections, (iv) serious infections, (v) upper respiratory tract infections (URTIs), (vi) gastroenteritis, (vii) autoimmune reactions, and (viii) dermatologic AEs (Supplementary Table 1). To maintain consistency across studies, only the incidence (proportion or number of patients with AE) of AEs were captured. Rate data (events/patient-year or total number of events) was not captured.

### Systematic Review and Analyses for Efficacy and Safety

#### Efficacy Endpoints

Raw proportions (%) in each study arm for ACR Pedi 30, 50, and/or 70 responses 3 months after treatment initiation were recorded or calculated as the number of subjects with response divided by the total number of subjects evaluable for response. RRs were calculated as % response in bDMARD or JAK inhibitor arm divided by % response in control arm. Further, 95% confidence intervals (CIs) for RRs were computed utilizing the exact method. All CIs that did not include 1 indicated significant effects. The control arm was defined as (i) placebo or (ii) no treatment on top of standard of care (SOC). SOC in the analyzed RCTs included background treatment with non-steroidal anti-inflammatory drugs, corticosteroids, and methotrexate. RRs of Pedi ACR 30, 50, and 70 3 months after treatment initiation were visualized using forest plots.

The pooled RR for each outcome was calculated using the DerSimonian-Laird method with a random effects model. Heterogeneity between studies was assessed using I^2^ (the proportion of variability between studies due to heterogeneity). Heterogeneity was defined by the following I^2^ thresholds: no heterogeneity I^2^ = 0%, low I^2^ < 30%, moderate 30 ≤ I^2^ ≤ 59%, and high I^2^ ≥ 60%. Since the meta-analysis for each ACR Pedi outcome included less than 10 studies, statistical significance of heterogeneity was assessed as *p* < 0.10. Meta-regression was also not performed due to the number of studies.

#### Safety Endpoints

The incidence of each AE of interest (see section “Safety Data”) was summarized descriptively for each arm at the end of each study in addition to calculating the risk difference (RD; % AE in bDMARD/JAK inhibitor arm - % AE in control arm).

#### Publication Bias and Software Package

Publication bias was assessed using visual inspection of the funnel plots; asymmetry of the funnel plots was assessed using Begg’s test (rank correlation method) and Egger’s test (linear regression method). Statistical significance for publication bias was assessed as *p* < 0.05. Meta-analyses were performed using the “meta” package in R (version 4.0.3). All forest plots and other graphs were performed using RStudio (version 1.2.5042).

## Results

Out of the 41 previously identified RCTs, 28 were performed in JIA/JIA-uveitis patients. Of these 28 RCTs, 23 reported Pedi ACR 30/50/70 at any time point, while 9 parallel design studies reported Pedi ACR 30/50/70 3 months after treatment start (Figure 1 and Table 1). The later 9 RCTs all reported safety data and were included in this systematic review as well as three withdrawal studies meeting inclusion criteria. No studies with JAK inhibitors were identified for the efficacy analysis subset.

**TABLE 1:** Overview of ACR Pedi 30/50/70 data reporting across 28 JIA RCTs (February 2022).

JIA subgroup	Drug	Study	Pedi ACR 30/50/70	Month 3 data^&^	Other time points (weeks)	ACR Pedi criteria
PJIA	Adalimumab	DE038, NCT00048542^$^	Yes	No	32	Giannini et al. (8)
	Anakinra	990758–990779, NCT00037648^$^	No			
	Etanercept	16.0016, NCT03780959^$^	Yes	No	17.33	Giannini et al. (8)
	Etanercept	16.0028, NCT03781375	Yes	No	26	Giannini et al. (8)
	Etanercept + MTX + steroid	TREAT, NCT00443430	Yes^#^	No	4, 9, 17, 22, 26, 52	Giannini et al. (8)
	Etanercept + MTX	Alexeeva 2021	Yes	Yes	4, 8	Giannini et al. (8)
	Infliximab	CR004774, NCT00036374	Yes	Yes	2, 6	Giannini et al. (8)
	Infliximab + MTX	ACUTE-JIA, NCT01015547	Yes	Yes	6, 24, 36, 48, 54	Giannini et al. (8)
sJIA	Anakinra	ANAJIS, NCT00339157	Yes	No	4.33	Giannini et al. (8) + no fever + CRP criteria^%^
	Canakinumab	β-SPECIFIC 1, NCT00886769	Yes	No	2, 4	Giannini et al. (8) + no fever
	Canakinumab	β-SPECIFIC 2, NCT00889863^$^	Yes	No	88	Giannini et al. (8) + no fever
	Canakinumab	β-SPECIFIC 4, NCT02296424^$^	No			
	Etanercept	20021631, NCT00078806^$^	No			
	Rilonacept	RAPPORT, NCT00534495	Yes	Yes	2*, 4, 6*,8*,10*	Giannini et al. (8) + no fever^%^
	Rilonacept	IL1T-AI-0504, NCT01803321	Yes	No	4	Giannini et al. (8) + no fever
	Tocilizumab	MRA316JP, NCT00144599^$^	Yes	Yes	2, 4, 6, 8, 10	Giannini et al. (8) + CRP criteria^%^
	Tocilizumab	TENDER, NCT00642460	Yes	Yes		Giannini et al. (8) + no fever^%^
ERA	Adalimumab	M11-328, NCT01166282	Yes	Yes		Giannini et al. (8)
	Adalimumab	HUM06-037, EudraCT 2007–003358-27	Yes^##^	Yes	4, 8	Giannini et al. (8)
	Etanercept	REMINDER, EudraCT 2010–020423-51^$^	No			
Mixed JIA (PJIA, sJIA^a^, eOJIA)	Abatacept	IM101-033, NCT00095173^$^	Yes	No	26	Giannini et al. (8)
(PJIA, OJIA, PsA)	Etanercept + MTX	BeSt for Kids, NTR1574	Yes	Yes	6, 26, 29, 52, 65, 78, 91, 104	Giannini et al. (8)
(PJIA^b^, eOJIA, PsA, sJIA)	Golimumab	GO KIDS, NCT01230827^$^	Yes	No	32	Giannini et al. (8)
(PsA, ERA)	Secukinumab	JUNIPERA, NCT03031782^$^	Yes	No	104	Giannini et al. (8)
(PJIA, eOJIA)	Tocilizumab	CHERISH, NCT00988221^$^	Yes	Yes*	4*, 8*, 16*, 20*, 24	Giannini et al. (8)
(PJIA, PsA, ERA)	Tofacitinib	A3921104, NCT02592434^$^	Yes	Yes	2, 6, 10, 18, 22, 26	Giannini et al. (8)
JIA-uveitis	Adalimumab	ADJUVITE, NCT01385826	No			
	Adalimumab	SYCAMORE, EudraCT 2010–021141-41	Yes	Yes	4, 9, 26, 39, 52, 65, 78	Giannini et al. (8)

*ERA, enthesitis-related juvenile idiopathic arthritis; eOJIA, extended oligoarticular juvenile idiopathic arthritis; JIA, juvenile idiopathic arthritis; MTX, methotrexate; PJIA, polyarticular juvenile idiopathic arthritis; PsA, psoriatic juvenile idiopathic arthritis; sJIA, systemic juvenile idiopathic arthritis.*

*^a^Without systemic features.*

*^b^RF + and RF-.*

*^&^Weeks 12–14.*

*^#^ACR Pedi 70 only.*

*^##^Does not include ACR Pedi 50.*

*ACR Pedi 30 only.

*^%^Also reports unmodified Giannini et al. (8) criteria.*

*^$^Withdrawal study design.*

In the efficacy analysis, of the 9 RCTs, 3 were conducted in PJIA, 2 in sJIA, 2 in ERA, 1 in JIA-uveitis, and 1 was performed in a conglomerate of several JIA subgroups (Table 1). A total of 6 studies compared bDMARD arms with placebo, and 3 studies compared with methotrexate monotherapy. In the safety analysis, of the 12 RCTs, 3 were conducted in PJIA, 3 in sJIA, 2 in ERA, 1 in JIA-uveitis, and 3 were performed in a conglomerate of several JIA subgroups (Table 1). A total of 9 studies compared bDMARD or JAK inhibitor arms with placebo, and 3 studies compared with methotrexate monotherapy. In the Alexeeva (20) study, etanercept combined with methotrexate was compared against methotrexate monotherapy; in the ACUTE-JIA study, infliximab combined with methotrexate was compared against methotrexate monotherapy; and in the BeSt for Kids study, the control arm was methotrexate or sulfasalazine. In the RAPPORT study, the control arm was placebo for the first 4 weeks and then switched to rilonacept until the end of the study. Two non-bDMARD, non-control arms were excluded from analysis: one with methotrexate combined with sulfasalazine and hydroxychloroquine in the ACUTE-JIA study, and one with methotrexate combined with prednisolone in the BeSt for Kids study.

### Systematic Review for Efficacy

**FIGURE 3:**
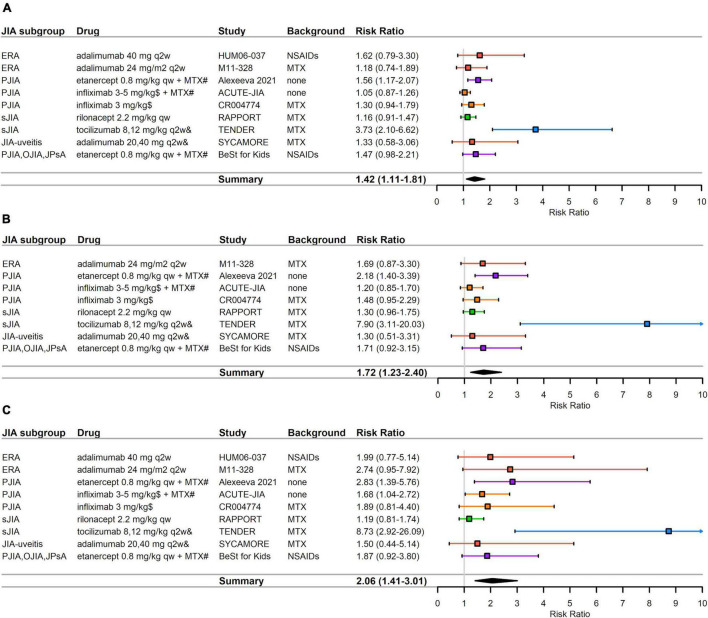
RCTs that reported efficacy data at month 3 (e.g., weeks 12–14). **(A)** ACR Pedi 30 data **(B)** ACR Pedi 50 data **(C)** ACR Pedi 70 data. RRs—mean represented by the square—were calculated as % response in bDMARD or JAK inhibitor arm divided by % response in control arm. Overall effect estimate is represented by the diamond, which width shows the confidence intervals for the overall estimated effect estimate. Further, 95% CIs for RRs—represented by the whiskers—were computed utilizing the exact method. All CIs that did not include 1 indicated significant effects. Experimental treatment is preferred when RR > 1. ACR Pedi, American College of Rheumatology pediatric responses; eOJIA, extended oligoarticular juvenile idiopathic arthritis; ERA, enthesitis-related juvenile idiopathic arthritis; JIA, juvenile idiopathic arthritis; MTX, methotrexate; NSAID, non-steroidal anti-inflammatory drugs; PJIA, polyarticular juvenile idiopathic arthritis; PsA, psoriatic juvenile idiopathic arthritis; qw, every week; q2w, every 2 weeks; q4w, every 4 weeks; q6w, every 6 weeks; sJIA, systemic juvenile idiopathic arthritis; RR, risk ratio. ^#^Compared against methotrexate monotherapy instead of placebo. ^$^Weeks 0,2,6,q6w for ACUTE-JIA study and weeks 0,2,6 for CR004774 study. ^&^8 mg/kg for > 30 kg, 12 mg/kg for < 30 kg for tocilizumab in TENDER study; 20 mg for < 30 kg, 40 mg for > 30 kg for adalimumab in the SYCAMORE study.

The 9 RCTs that reported ACR Pedi 30/50/70 responses at 3 months evaluated different bDMARDs and JAK inhibitors in various JIA subgroups (Table 2). Two studies evaluated adalimumab in ERA patients and one study in JIA-uveitis patients. Two studies evaluated infliximab, and one etanercept in PJIA. One study with tocilizumab and one with rilonacept were conducted in sJIA patients, while one study with etanercept was conducted in a conglomerate of several JIA subgroups at 3 months. All treatment arms with bDMARDs or JAK inhibitors showed improved ACR Pedi responses over control arms. RRs ranged from 1.05 to 3.73 in ACR Pedi 30, from 1.20 to 7.90 in ACR Pedi 50, and from 1.19 to 8.73 in ACR Pedi 70. The HUM06-037 study reported Pedi ACR 30 and 70 but not Pedi ACR 50 (Table 2). There was high and statistically significant heterogeneity in the meta-analysis of all three ACR Pedi outcomes (I^2^ = 75% and *p* < 0.01 for ACR Pedi 30; I^2^ = 71% and *p* < 0.01 for ACR Pedi 50; and I^2^ = 58% and *p* = 0.01 for ACR Pedi 70) (Figure 3).

**TABLE 2:** Overview of Pedi ACR 30/50/70 data at 3 months (e.g., weeks 12–14) in JIA RCTs.

JIA subgroup	Drug	Study	Number of included patients		Pedi ACR 30^&^	Pedi ACR 50^&^	Pedi ACR 70^&^
			bDMARD arm	Control arm			
ERA	Adalimumab	HUM06-037	17	15	65| 40%	NA	53| 27%
	Adalimumab	M11-328	31	15	71| 60%	68| 40%	55| 20%
PJIA	Etanercept + MTX^#^	Alexeeva (20)	35	33	97| 62%	85| 40%	59| 22%
	Infliximab + MTX^#^	ACUTE-JIA	19	20	94| 90%	84| 70%	84| 50%
	Infliximab	CR004774	58	59	64| 49%	50| 34%	22| 12%
sJIA	Rilonacept	RAPPORT	33	29	88| 76%	85| 66%	70| 59%
	Tocilizumab	TENDER	75	37	91| 24%	85| 11%	71| 8%
JIA-uveitis	Adalimumab	SYCAMORE	60	30	27| 20%	22| 17%	15| 10%
PJIA, OJIA, PsA	Etanercept + MTX^#^	BeSt for Kids	30	32	73| 50%	53| 31%	47| 25%

*bDMARD, biologic disease-modifying antirheumatic drug; eOJIA, extended oligoarticular juvenile idiopathic arthritis; ERA, enthesitis-related juvenile idiopathic arthritis; JIA, Juvenile idiopathic arthritis; NA, Not available; PJIA, Polyarticular juvenile idiopathic arthritis; PsA, Psoriatic juvenile idiopathic arthritis; sJIA, systemic Juvenile idiopathic arthritis.*

*^&^Proportion of patients in bDMARD arm| proportion of patients in control arm with response.*

*^#^Compared against methotrexate monotherapy instead of placebo.*

The TENDER study lies outside the funnel plots (Figure 2) for all three ACR Pedi outcomes and contributes the most heterogeneity (data not shown). Funnel plot asymmetry was significant by Egger’s test for Pedi ACR 70 (*p* = 0.035) but not significant for ACR Pedi 30 (*p* = 0.068) or ACR Pedi 50 (*p* = 0.091). Funnel plot asymmetry was not significant by Begg’s test for ACR Pedi 30 (*p* = 0.297), ACR Pedi 50 (*p* = 0.216) or Pedi ACR 70 (*p* = 0.211). Due to the low number of studies, results must be interpreted with caution as tests for publication bias are underpowered when there are less than 10 studies in an analysis.

#### Pedi ACR 30

Significant treatment responses vs. placebo were observed for tocilizumab in sJIA. The RR for tocilizumab was 1.48 (95% CI 1.03–2.13) in the MRA316JP study, and 3.73 (95% CI 2.10–6.62) in the TENDER study. In addition, tofacitinib showed superior efficacy vs. placebo in PJIA with RR = 1.45 (95% CI 1.12–1.87) (Figure 3A and Table 2). Etanercept combined with methotrexate showed superior efficacy compared to methotrexate monotherapy in PJIA with RR = 1.56 (95% CI 1.17–2.07). The pooled RR for Pedi ACR 30 was 1.42 (95% CI 1.11–1.81), indicating in general bDMARD therapy is significant superior compared to placebo or methotrexate (Figure 3A).

#### Pedi ACR 50 and ACR 70

Significant treatment responses compared with placebo were observed for Pedi ACR 50 and Pedi ACR 70 in the same studies as for Pedi ACR 30 (Figures 1B,C and Table 2). The RRs for tocilizumab in the MRA316JP study were 1.60 (95% CI 1.09–2.36) for Pedi ACR 50 and 2.47 (95% CI 1.37–4.47) for Pedi ACR 70. The RRs for tocilizumab in the TENDER study were 7.90 (95% CI 3.11–20.03) for Pedi ACR 50 and 8.73 (95% CI 2.92–26.09) for Pedi ACR 70. As for tofacitinib, the RRs in the A3921104 study were 1.57 (95% CI 1.16–2.13) for Pedi ACR 50 and 1.48 (95% CI 1.03–2.11) for Pedi ACR 70. An enhanced effect for ACR Pedi 70 was also observed with infliximab plus methotrexate in PJIA (RR = 1.68, 95% CI 1.04–2.72) compared to methotrexate monotherapy in the ACUTE-JIA study. Similar superior efficacy was observed for etanercept combined with methotrexate compared to methotrexate monotherapy in PJIA. The pooled RR for Pedi ACR 50 was 1.72 (95% CI 1.23–2.40) (Figure 3B) and for Pedi ACR 70 was 2.06 (95% CI 1.41–3.01) (Figure 3C).

### Systematic Review for Safety

**FIGURE 4:**
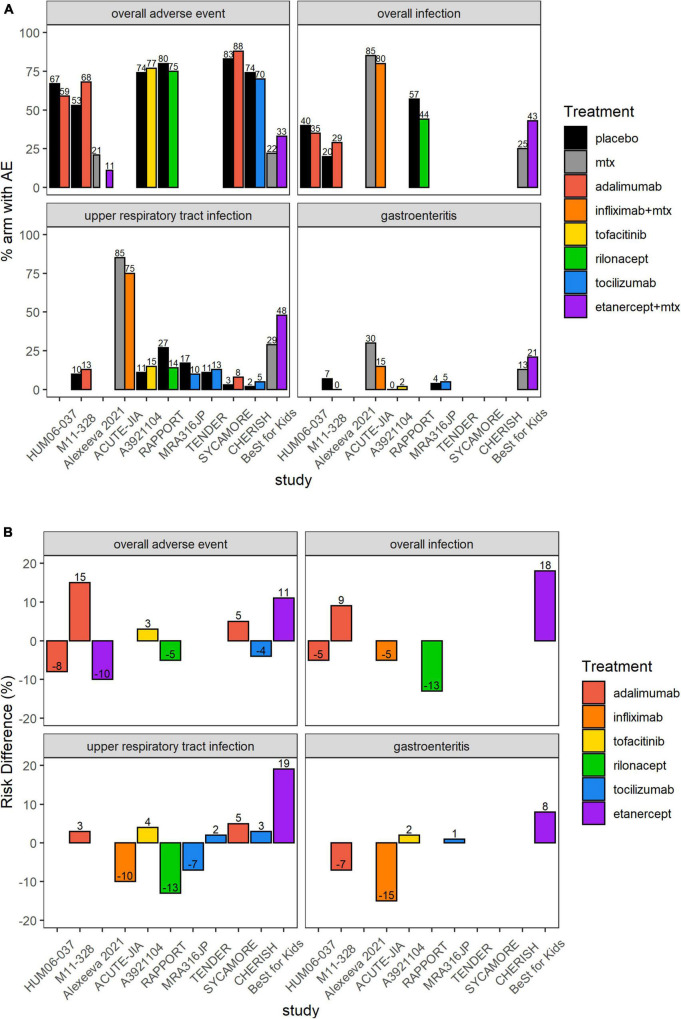
Overall AEs, overall infections, upper respiratory infections or gastroenteritis in the 12 included RCTs. **(A)** Proportion (%) of patients with AEs in each study arm. Higher proportion of AEs in the placebo or SOC arm favors experimental treatment. **(B)** RD (%) of patients with AEs in each treatment arm compared to placebo or SOC. Treatment effect < 0 favors experimental treatment over placebo or SOC. AE, adverse event; MTX, methotrexate; SOC, standard of care. In the ACUTE-JIA study, the rate of overall AEs was 4.8 events/patient-year in the infliximab plus methotrexate arm and 6.5 events/patient-year in the methotrexate monotherapy arm. In the TENDER study, the rate of overall AEs was 9.3 events/patient-year in the tocilizumab arm and 9.4 events/patient-year in the placebo arm; corresponding rates of overall infections were 3.4 vs. 2.9 events/patient-year.

Figure 4 shows an overview of the frequency of overall AEs, overall infections, URTIs, and gastroenteritis in the 12 studies that reported ACR Pedi 30, 50, or 70 after 3 months of treatment. The incidence of overall AEs and URTIs appeared to be more frequent with adalimumab in JIA-uveitis (88 vs. 83% and 8 vs. 3%, respectively), and with adalimumab in ERA in the M11-328 study (68 vs. 53% and 13 vs. 10%, respectively) compared to control arms. Gastroenteritis was reported more frequently with etanercept (21 vs. 13%) than in control arms. SAEs were more frequently reported with adalimumab in JIA-uveitis (22 vs. 7%), with adalimumab in ERA (12% vs. 7% for the HUM06-037 study, and 3 vs. 0% for the M11-318 study), and with rilonacept in sJIA (8 vs. 3%) compared to control arms. The incidence of serious infections was higher with adalimumab in JIA-uveitis (13 vs. 0%) compared to control arm. The incidence of infections was considerably higher in the ACUTE-JIA (PJIA) study: 80 vs. 85% for overall infections, 75 vs. 85% for URTIs, and 15 vs. 30% for gastroenteritis in the infliximab-methotrexate arm and methotrexate monotherapy arm, respectively.

**FIGURE 5:**
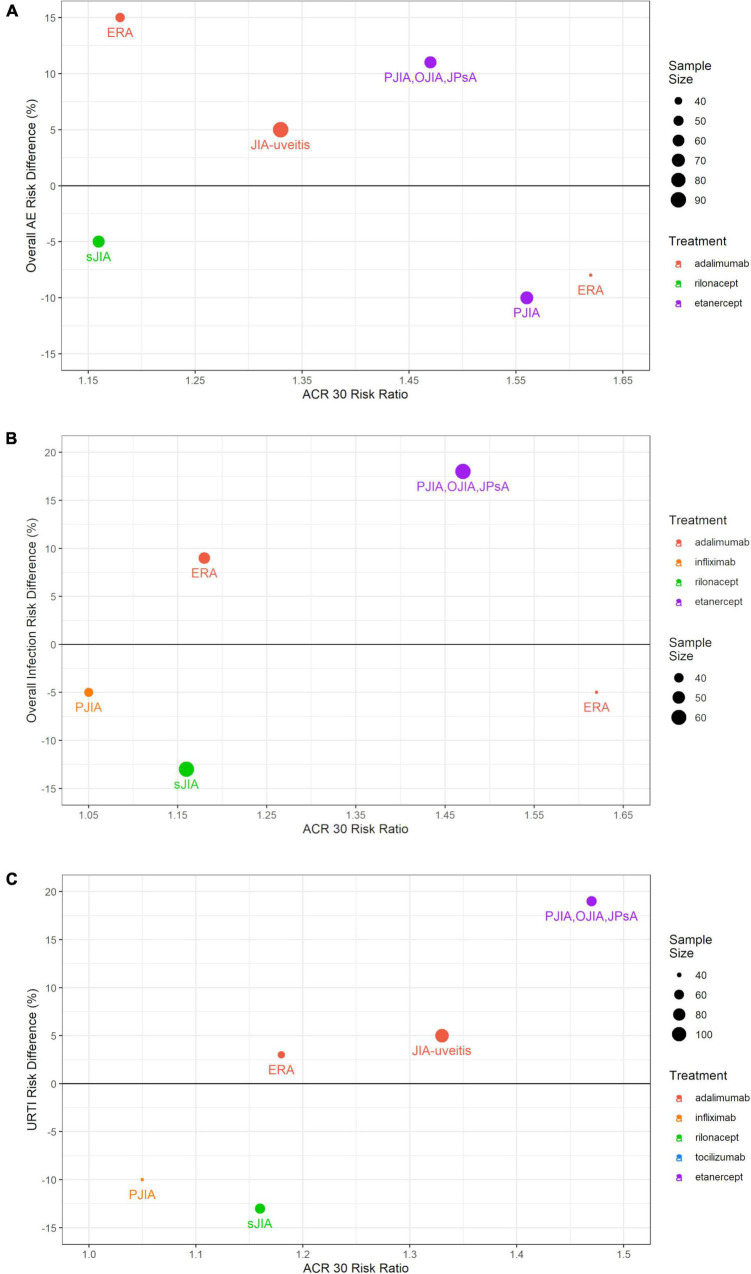
Comparing efficacy (ACR Pedi 30 RRs) against safety (RD) in the RCTs that reported both outcomes. **(A)** ACR Pedi 30 RR vs. any AE RD; **(B)** ACR Pedi 30 RR vs. any infection RD; **(C)** ACR Pedi 30 RR vs. URTI RD. The size of each dot corresponds to the sample size. The color of each dot corresponds to the treatment used in the studied arm. ACR Pedi, American College of Rheumatology pediatric responses; RCT, randomized controlled trial; RD, risk difference; URTI, upper respiratory tract infection.

Table 3 provides an overview of predefined AEs in the 12 RCTs that reported ACR Pedi outcomes at month 3. The reporting of AEs in the CR004774 study created an unbalanced evaluation of the two arms and was therefore not recorded for this analysis. In this study, PJIA patients were randomized to either the experimental arm of infliximab 3 mg/kg combined with methotrexate for 44 weeks, or the control arm with methotrexate monotherapy for 6 weeks followed by infliximab 6 mg/kg combined with methotrexate until week 44. The safety data for the control arm was reported separately for weeks 0–14 and 14–52, but for the active arm for weeks 14–52. The lack of separate reporting for weeks 0–14 for the active arm makes it challenging to directly compare infliximab against placebo for the first fourteen weeks of the study. Of the other 11 studies that reported both efficacy and safety data, the most frequently reported AEs were respiratory tract infections such as URTI and bronchopneumonia, with higher numbers in bDMARD or JAK inhibitor arms than in control arms (Table 3). Few dermatologic AEs, such as injection-site or infusion reactions, were reported. However, it should be noted that injection-site reactions were reported in 8 patients treated with adalimumab, but in no patients treated with placebo in the SYCAMORE JIA-uveitis trial. Anti-drug antibodies were reported in one tocilizumab RCT. Weighing infection risk and therapeutic effects of bDMARDs and JAK inhibitors against each other in therapeutic management of patients with JIA is important. Therefore, bDMARDs and JAK inhibitors in PJIA and various subgroups of JIA were compared for efficacy (ACR Pedi 30 RRs) against safety (RD) (Figure 5). As an example, etanercept treatment in mixed JIA was associated with increased risk of any infection while increasing achievement of ACR Pedi 30 by ∼50% as compared to control arms (Figure 5B). Overall, no unexpected safety outcomes were found in this analysis.

**TABLE 3:** Overview of adverse events of interest in the 12 JIA RCTs that reported Pedi ACR 30/50/70 data at month 3 (i.e., weeks 12–14).

JIA subgroup	Drug	Study	bDMARD arm	Control arm	Time (weeks)	Allergic or autoimmune reactions%	Dermatologic reactions%	Respiratory infections%	Gastrointestinal or hepatic reactions%
ERA	Adalimumab	HUM06-037	17	15	12	Injection site reaction (3|4)&			
	Adalimumab	M11-328	31	15	12	Injection site erythema (1|0), injection site pain (3|1)	Paronychia* (1|1)	Bronchopneumonia* (1|0), nasopharyngitis* (0|1), pharyngitis* (1|0), sinusitis* (1|0), URTI* (3|2)	Any hepaticae (1|0), gastroenteritis (2|0), hepatocellular injury (1|0)
PJIA	Etanercept+ MTX#	Alexeeva 2021	35	33	48			Acute respiratory infection (1|0), rhinitis (0|1)	
	Infliximab + MTX#	ACUTE-JIA	20	20	54	Antinuclear antibodies newly positive (1|0), transient infusion-related reaction (2|0)		URTI (15|17)	Gastroenteritis (3|6)
	Infliximab	CR004774	60	62	14				
	Tofacitinib	A3921104	88**	85**	26			Nasopharyngitis* (7|3), pharyngitis* (2|1), pharyngitis streptococcal* (2|0), RTI* (3|1), RTI viral* (1|2), rhinitis* (2|1), sinusitis* (4|1), tonsillitis* (1|2), URTI* (13|9)	Appendicitis serious (0|1), gastroenteritis* (2|0)
SJIA	Rilonacept	RAPPORT	35	33	24	Injection site reaction (2|3)		URTI* (5|9), pharyngitis streptococcal* (2|2)	
	Tocilizumab	MRA316JP	21	23	12	1*^a^*		URTI (2|4)	Gastroenteritis (1|1)
	Tocilizumab	TENDER	75	37	12	Antidrug antibodies (1|1)		Pharyngitis (10|3), URTI (10|4)	
JIA-uveitis	Adalimumab	SYCAMORE	60	30	78	Injection site reaction (8|0)	impetigo* (4|1), molluscum contagiosum (2|0), paronychia* (3|1), skin infection*(2|0), skin papilloma* (5|0)	LRTI* (8|2), nasopharyngitis (17|8), pharyngitis* (4|0), pneumonia serious (1|0), rhinitis* (2|1), tonsillitis* (13|0), tonsilitis serious (1|0), tonsilitis streptococcal* (1|0), URTI* (5|1), LRTI serious (1|0), streptococcal infection (2|0)	
PJIA, eOJIA	Tocilizumab	CHERISH	82	81	24	2*^b^*		Pharyngitis (3|3), URTI (4|2), nasopharyngitis (14|9), pneumonia (1|0), rhinitis (2|1)	Gastroenteritis (0|1) leading to study discontinuation
PJIA, OJIA, PsA	Etanercept+ MTX#	BeSt for Kids	30	32	104			URTI (14|9)	Gastroenteritis (6|4)

*AE = adverse event; eOJIA = extended oligoarticularjuvenile idiopathic arthritis, ERA = enthesitis-related juvenile idiopathic, JIA = juvenile idiopathic arthritis, LRTI = lower respiratory tract infection, MTX= methotrexate, PJIA = polyarticularjuvenile idiopathic arthritis, PsA = psoriatic juvenile idiopathic arthritis; RTI = respiratory tract infection, sJIA = systemic juvenile idiopathic arthritis, and URTI = upper respiratory tract infection.*

*^#^Compared against methotrexate monotherapy instead of placebo.*

*Specified as nonserious AE.

*^&^Reported as general disorders and administration site conditions.*

*^%^Number of patients in bDMARD arm | number of patients in control arm.*

**Efficacy data were reported only in PJIA subgroup, but safety data were reported across all JIA subgroups (72 PJIA/7 PsA/9 ERA in tofacitinib arm and 70 PJIA/8 PsA/7 ERA in placebo arm).

*^a^For the MRA316JP study, sources stated that 4 patients developed antidrug antibodies and 10 patients developed mild infusion reactions but it was not clarified that it was in the tocilizumab arm.*

*^b^For the CHERISH study, two patients had positive neutralizing antidrug antibodies, but it was not specified that it was in the tocilizumab arm.*

## Discussion

This systematic review expands on previous systematic reviews that evaluated benefits and risks of bDMARDs in JIA patients (21–24). In line with previous data syntheses, the aggregate data of included RCTs indicated superior treatment responses compared to placebo or SOC (24–26). Surprisingly, the lower limit of the RR CIs for the ACR Pedi 30, 50, and 70 in these trials is frequently less than one, although all the proportion of bDMARD-treated patients achieving ACR Pedi responses exceeded the placebo-treated or methotrexate only treated patients. The magnitude of treatment response was more pronounced with ACR Pedi 50 and 70 vs. ACR Pedi 30. No unexpected safety signals were found in this analysis.

### Efficacy Outcomes With Biologic Disease-Modifying Antirheumatic Drugs or Janus Kinase Inhibitors

All analyzed RCTs used ACR Pedi response criteria to define improvement in disease activity, as these criteria seem to be the gold standard for assessing treatment responses (9). Previously published treatment recommendations highlighted the importance of regular disease activity assessments, for example with the Juvenile Arthritis Disease Activity Score (JADAS) to adapt treatment (27). Treatment aims to reach at least a 50% improvement in disease activity within 3 months, and within 6 months patients should reach the defined target (e.g., remission) and treatment should be adjusted until target is achieved (27). Therefore, treatment efficacy should be assessed on a regular basis, e.g., every 3 months (28). Previous analyses have shown that the maximum clinical benefit of bDMARDs (expressed in percentages of improvement) is not achieved before 3 months of treatment (27). This might be explained at least in part by the fact that 3 months of treatment is close to the time to reach steady state (corresponding to 5 half-lives, with terminal half-lives being between 2 and 3 weeks) for commonly used bDMARDs.

Based on established knowledge of the pharmacokinetics and in line with recommendations, this analysis compared ACR Pedi data 3 months after treatment initiation as this is a critical timepoint during patient care. Interestingly, fewer than half of all included RCTs in this systematic review (12 out of 28 RCTs) assessed efficacy after 3 months of treatment. All 12 clinical studies reported ACR Pedi 30 responses, which is the commonly and historically used outcome measurement in clinical trials for regulatory approval of new therapies (9, 27). A total of 11 studies reported ACR Pedi 50 or ACR Pedi 70, although ACR Pedi 50 corresponds to the treatment response target after 3 months of treatment in JIA. ACR Pedi 90 was reported in seven of these 12 clinical studies in children. In the meta-analysis, only 9 RCTs remained after taking out withdrawal designs for ACR Pedi 30 and 70 and 8 RCTs for ACR Pedi 50. This suggests that different scales were investigated in previously published pediatric RCTs. While these results show a comprehensive comparison of treatment responses to various bDMARDs, it should be noted that several JIA studies were excluded as they did not fulfill inclusion criteria. Remission and minimal disease activity is associated with prevention of disease-related damage and is nowadays the target in daily clinical practice. Therefore, it would be desirable that all future RCTs in JIA report ACR Pedi 50, 70, and 90 after 3 and 6 months.

### Safety Outcomes With Biologic Disease-Modifying Antirheumatic Drugs and Janus Kinase Inhibitors

There is evidence suggesting that uncontrolled rheumatic inflammatory disease itself can increase the risk of infection (29, 30). Incidence of bacteremia has been reported to be three times higher in children with JIA compared to the general pediatric population (29). In particular, high disease activity seems to be associated with an elevated infection risk (30, 31). Furthermore, uncontrolled disease activity has the risk of resulting in organ damage. In JIA, articular damage is a dreaded complication in untreated patients, particularly in polyarthritis. In addition, ocular sequelae due to uncontrolled disease activity of uveitis is a major concern in children with JIA. Moreover, uncontrolled disease activity leads to decreased health-related quality of life and might result in limited social participation. Socially restricted patients tend to have a higher degree of disability and lower levels of physical functioning, self-esteem, and emotional wellbeing (32). Therefore, treatment in JIA is crucial, and in most JIA patients immunosuppressive treatment with bDMARDs or JAK inhibitors is required to achieve inactive disease or lowest possible disease activity to avoid disease complications. However, immunosuppressive treatments can increase the risk of infections depending on treatment duration, route of administration, drug, and dosage (33–35). In any case, a higher susceptibility to infections associated with bDMARDs or JAK inhibitors should be weighed against the therapeutic effects in children with JIA (Figure 5). To further illustrate this point, etanercept treatment in mixed JIA was associated with increased risk of any infection while increasing achievement of ACR Pedi 30 by ∼50% as compared to control arms (Figure 5B). Overall, no unexpected safety outcomes were found in this systematic review and treatment with bDMARDs appears to be safe. Long-term safety outcomes could not be assessed in this study as investigated RCTs reported safety data up to 2 years only. It should be noted that in the past, several registries have focused on long-term observations in children treated with bDMARDs.

### Drug Development in Children With Juvenile Idiopathic Arthritis

As previously reported, multiple bDMARDs and JAK inhibitors such as abatacept, anakinra, rituximab, sarilumab, tocilizumab, adalimumab, certolizumab pegol, etanercept, golimumab, infliximab, baricitinib, tofacitinib, and upadacitinib are approved for adults with RA (12). In contrast, for children with JIA the number of approved bDMARDs and JAK inhibitors is limited (12). While the statement that children are not small adults has been known for years, the same therapeutic armamentarium of bDMARDs is usually prescribed in pediatric and adult rheumatology, and off-label dosing regimens are carried out relying on existing adult data (17). However, the disease course differs between pediatric and adult rheumatology patients, and pharmacokinetic processes undergo significant changes during growth and development (36). While a plethora of clinical trial results is available on dosing biologics and associated efficacy and safety in adult rheumatology, only a limited number of studies have analyzed pharmacokinetics, pharmacodynamics, and clinical efficacy and safety outcomes in children with JIA. Further, RCTs in adults with RA may investigate multiple dose levels allowing characterization of dose-response relationships, whereas clinical studies in children with JIA tend to investigate one dose level only. Another noticeable difference between adults with RA and children with JIA is that there are multiple subtypes of JIA with different clinical responses, making it even more challenging to understand and standardize treatment recommendations for children suffering from PiRDs (12).

Exploratory and model-based meta-analysis is a powerful tool corresponding to the statistical practice of combining large amounts of data from different trials to generalize or strengthen the findings (37). Model-based meta-analysis (MBMA) can accelerate drug development by informing and enhancing key decisions in drug development (38, 39). MBMA allows for the compilation of clinical responses across drugs that is facilitated by the integration and utilization of summary-level efficacy and safety data across different treatments, providing a quantitative framework for comparative efficacy and safety assessment (40, 41). One major difference between systematic review and MBMA is that the latter explicitly incorporates the effect of dose and duration using standard pharmacology models and assumptions, allowing dose–response relationships to be characterized as well as the impact of covariates on the dose–response relationships (42). The incorporation of adult information as well as the use of optimization techniques in MBMA could increase parameter precision in pediatric rheumatology (43). As such, literature review and meta-analyses can support development of new treatments in pediatric rheumatology, by providing quantitative tools to bridge adult and pediatric clinical outcomes data and better characterize and compare the efficacy-safety balance of existing and new bDMARDs and JAK inhibitors in vulnerable children with a PiRD such as JIA.

### Limitations

There are several limitations of this systematic review, including the relatively small sample size of the studies and number of therapeutic entities reported in previously published RCTs. All studies were reported independent on study design for safety analysis. As three studies were conducted with withdrawal designs, they were excluded from the later performed pooled analysis on efficacy data adjusted for sample size to avoid overestimation of treatment effect. This further reduced the number of RCTs included in the meta-analysis, which results had to be interpreted with caution. Firstly, follow-up duration was not the same for every bDMARD. RCTs included in this study showed high-quality data, and strong RCT selection criteria were used while performing the literature search. Secondly, there were significant variations in the reporting of safety across different JIA RCTs. Most RCTs reported proportions, while others reported AE rates. A majority of investigated studies reported safety endpoints at the end of each study (i.e., various time points across investigated studies in this analysis). We therefore interpret reported safety data with caution. Of course, standardization of safety outcome measures in pediatric studies would facilitate direct comparison between studies and patient-level meta-analysis.

## Conclusion

Investigated bDMARDs and JAK inhibitors showed superior treatment responses compared to controls after 3 months of treatment, which were more pronounced in ACR Pedi 50 and 70 vs. ACR Pedi 30. Higher susceptibility to infections was observed with bDMARDs or JAK inhibitors as compared to placebo or methotrexate monotherapy. Such safety outcomes should be weighed against disease-related damage and risk of decreased health-related quality of life due to uncontrolled disease activity. Additional clinical studies are warranted to further inform development and utilization of biologics to further enhance treat-to-target strategies, therapeutic management, and overall patient care in juvenile idiopathic arthritis.

## Author Contributions

KG and CW performed the systematic review of efficacy and safety data and were responsible for execution and documentation. TW provided support as therapeutic area expert. Any discrepancies were resolved through discussion or consultations with a third independent reviewer MP. KG, CW, CY, NZ, TW, and MP contributed to the preparation of the submitted manuscript. All authors were involved in designing and critically revising the research project, approved this version to be published, and they agreed to be accountable for all aspects by ensuring questions related to the accuracy or integrity of any part of the work were appropriately investigated and resolved, agreed to the submission of this manuscript to frontiers.

## Conflict of Interest

CW, CY, NZ, and MP were employed by Certara LP. The remaining authors declare that the research was conducted in the absence of any commercial or financial relationships that could be construed as a potential conflict of interest.

## Publisher’s Note

All claims expressed in this article are solely those of the authors and do not necessarily represent those of their affiliated organizations, or those of the publisher, the editors and the reviewers. Any product that may be evaluated in this article, or claim that may be made by its manufacturer, is not guaranteed or endorsed by the publisher.
