# Supplementary material for: Value of Literature Review to Inform Development and Use of Biologics in Juvenile Idiopathic Arthritis
Source: Front Pediatr. 2022 Jun 21;10:909118. doi: 10.3389/fped.2022.909118 (PMC9253535; doi:10.3389/fped.2022.909118)
Supplement: Supplementary file 1 [file Table_1.docx]

**Supplementary material**

Review protocol

**Background**

This literature search was performed initially on July 26, 2020 and updated most recently on February 3, 2022 to analyze knowledge from randomized control trails (RCTs) in children with paediatric inflammatory rheumatic diseases (PiRD) treated with defined biologic disease modifying antirheumatic drugs (bDMARDs) and Janus kinase (JAK) inhibitors. The literature search was done systematically, based on relevant identification, screening, and assessment steps described in the Cochrane Handbook for Systematic Reviews of Interventions (1) and reporting items in the PRISMA statement (2).

For analysis performed in the manuscript only parallel-design RCTs performed in juvenile idiopathic arthritis (JIA) and JIA-uveitis with ACR Pedi responses 30/50/70 12-14 weeks after the start of treatment were of interest.

**Objective**

The primary objective of the literature search is to provide an independent depository of performed RCTs in PiRD patients treated with bDMARDs and JAK inhibitors.

**Methods**

1. *Search terms*

PubMed: (pediatric OR children OR infants OR juvenile OR neonatal OR teen OR adolescent) AND (abatacept OR adalimumab OR anakinra OR baricitinib OR belimumab OR brodalumab OR canakinumab OR certolizumab OR etanercept OR golimumab OR guselkumab OR infliximab OR ixekizumab OR risankizumab OR rilanocept OR rituximab OR sarilumab OR secukinumab OR tildrakizumab OR tocilizumab OR tofacitinib OR upadacitinib OR ustekinumab OR Janus kinase OR JAK inhibitor) AND randomized controlled trial [publication type] AND English

Clinicaltrials.gov search terms: For each drug listed in the inclusion criteria, the following search filter was applied: Interventional Studies (Clinical Trials) AND Child (birth-17) AND Studies with Results. To find recruiting studies, the “Studies with Results” filter was not included in the search terms.

1. *Eligibility*

Titles and abstracts are screened for eligibility according to inclusion and exclusion criteria:

Inclusion criteria:

- - Patients aged 20 years and younger
  - Treatment with predefined bDMARDs/JAK
    - Anti-CD20 agents: rituximab
    - CD80/86 inhibitors: abatacept
    - IL-1 inhibitors: anakinra, canakinumab, rilonacept
    - IL-6 inhibitors: tocilizumab, sarilumab;
    - IL-12/23 inhibitors: ustekinumab
    - IL-23 inhibitors: guselkumab, risankizumab, tildrakizumab
    - IL-17 inhibitors: secukinumab, ixekizumab, brodalumab
    - Tumour necrosis factor (TNF) inhibitors: adalimumab, etanercept, golimumab, infliximab, certolizumab pegol
    - BAFF inhibitors: belimumab
    - JAK inhibitors: baricitinib, tofacitinib, upadacitinib
  - Sample size ≥ five patients
  - Confirmed PiRD diagnosis
  - At least one relevant primary or secondary efficacy endpoint/outcome
  - English language
  - Published after 1990

Exclusion criteria

- - Indication not relevant
  - Population not relevant
  - Study design not relevant (not RCT)
  - Treatment not relevant
  - Endpoint/outcome not relevant
  - Duplicate of prior published results without any additional information

1. *Search sources and retrieval*

The primary sources of information for this database were PubMed, Cochrane Library, the US National Institutes of Health Ongoing Trials Register ClinicalTrials.gov ([www.clinicaltrials.gov](http://www.clinicaltrials.gov)), and the EU Clinical Trials Register ([www.clinicaltrialsregister.eu](http://www.clinicaltrialsregister.eu)) augmented by searches on conference abstracts/posters/presentations (ACR, EULAR, PRES, ISSAID), as well as regulatory reviews from FDA/EMA websites (www.fda.gov, www.ema.europa.eu).

1. *Identification*

Identification of eligible study reports were based on search terms. Web searches for company trial registries were conducted for additional data for drugs under development. Finally, references list of published studies were reviewed to identify any additional references. The search results were exported and managed in a spreadsheet and electronic copies (PDFs) of study reports are retrieved via internet sources or local libraries.

1. *Screening and eligibility assessment*

Initial screening, based on retrieved abstracts, as well as the eligibility assessment based on full-text publications were performed by two scientists. One scientist was responsible for the execution and documentation and the other provided support as the therapeutic area expert.

**Data extraction**

Data extraction was done by scientists using an electronic data extraction spreadsheet developed in MS Excel. Consensus sessions were held regularly to resolve data extraction issues.

**Final inclusion**

Final selection of the study reports was performed after the scientists involved in the project have a consensus. Our analysis in this manuscript focusing on safety data included RCTs in patients with JIA/JIA-uveitis with ACR Pedi responses 30/50/70. Analysis on efficacy data excluded withdrawal study designs.

***References***

1. Higgins JPT, Green S (editors). Cochrane Handbook for Systematic Reviews of Interventions Version 5.0.2 [updated September 2009]. The Cochrane Collaboration, 2009. Available from www.cochrane-handbook.org.
2. Moher D, Liberati A, Tetzlaff J, Altman DG, The PRISMA Group (2009). Preferred Reporting Items for Systematic Reviews and Meta-Analyses: The PRISMA Statement. PLoS Med 6(6): e1000097. doi:10.1371/journal.pmed1000097
