# Supplementary material for: Value of Literature Review to Inform Development and Use of Biologics in Juvenile Idiopathic Arthritis
Source: Front Pediatr. 2022 Jun 21;10:909118. doi: 10.3389/fped.2022.909118 (PMC9253535; doi:10.3389/fped.2022.909118)
Supplement: Supplementary file 2 [file Table_2.docx]

**Supplementary Table 1.** **Categorization of** **adverse events of interest in juvenile idiopathic randomized controlled trials**

| **AE category** | **Adverse Event** |
| --- | --- |
| **Allergic or autoimmune** | Allergic reaction  Anti-double strand DNA  Antidrug antibodies  Antinuclear antibodies  Drug hypersensitivity  Injection-site reaction |
| **Dermatologic** | Tinea corporis, including tinea pedis and tinea versicolor  Folliculitis  Fungal skin infection  Impetigo  Maculopapular rash  Molluscum contagiosum  Paronychia  Planar warts  Pustular rash  Skin infection  Skin papilloma |
| **Respiratory** | Bronchitis  Bronchopneumonia  Epiglottitis  Laryngitis  Lobar pneumonia  Lower respiratory tract infection  Nasopharyngitis  Pharyngitis, including streptococcal pharyngitis  Pharyngotonsillitis  Pleurisy  Pneumonia, including lobar pneumonia  Respiratory tract infection, including viral respiratory tract infection  Rhinitis  Sinusitis, including acute/chronic sinusitis  Stomatitis, including viral stomatitis  Tonsillitis, including acute/bacterial/streptococcal/viral tonsillitis  Tracheitis  Upper respiratory tract infection, including viral upper respiratory tract infection |
| **Gastrointestinal** | Appendicitis  Gastritis  Gastroenteritis, including viral gastroenteritis or salmonella gastroenteritis  Gastrointestinal infection, including viral gastrointestinal infection |
| **Hepatic** | Any hepatic adverse event  Hepatocellular injury  Hyperbilirubinemia  Sclerosing cholangitis |
